# Accuracy of tracheal aspirate gram stain in predicting *Staphylococcus aureus* infection in ventilator-associated pneumonia

**DOI:** 10.1186/1471-2253-15-19

**Published:** 2015-01-23

**Authors:** Renato Seligman, Beatriz Graeff Santos Seligman, Loriane Konkewicz, Rodrigo Pires dos Santos

**Affiliations:** Hospital de Clinicas de Porto Alegre, Rua Ramiro Barcelos 2350, 90035-003 Porto Alegre, Brazil; Universidade Federal do Rio Grande do Sul, Rua Ramiro Barcelos 2400 – 4o Andar, 90035-003 Porto Alegre, Brazil

**Keywords:** *Staphylococcus aureus*, Gram stain, Tracheal aspirate, Ventilator-associated pneumonia

## Abstract

**Background:**

The Gram stain can be used to direct initial empiric antimicrobial therapy when complete culture is not available. This rapid test could prevent the initiation of inappropriate therapy and adverse outcomes. However, several studies have attempted to determine the value of the Gram stain in the diagnosis and therapy of bacterial infection in different populations of patients with ventilator-associated pneumonia (VAP) with conflicting results. The objective of this study is to evaluate the accuracy of the Gram stain in predicting the existence of *Staphylococcus aureus* infections from cultures of patients suspected of having VAP.

**Methods:**

This prospective single-center open cohort study enrolled 399 patients from December 2005 to December 2010. Patients suspected of having VAP by ATS IDSA criteria were included. Respiratory secretion samples were collected by tracheal aspirate (TA) for standard bacterioscopic analysis by Gram stain and culture.

**Results:**

Respiratory secretion samples collected by tracheal aspirates of 392 patients were analyzed by Gram stain and culture. When Gram-positive cocci were arranged in clusters, the sensitivity was 68.4%, specificity 97.8%, positive predictive value 88.1% and negative predictive value 92.8% for predicting the presence of *Staphylococcus aureus* in culture (*p* < 0.001).

**Conclusions:**

A tracheal aspirate Gram stain can be used to rule out the presence of *Staphylococcus aureus* in patients with a clinical diagnosis of VAP with a 92.8% Negative Predictive Value. Therefore, 7.2% of patients with *Staphylococcus aureus* would not be protected by an empiric treatment that limits antimicrobial coverage to *Staphylococcus aureus* only when Gram positive cocci in clusters are identified.

## Background

Ventilator Associated Pneumonia (VAP) has high mortality rates [1, 2], and thus, early empirical prescription of broad spectrum antibiotics is mandatory for these patients [2, 3]. The trade-off for this practice is progressive induction of bacterial resistance in intensive care units (ICU). Aerobic Gram negative bacilli are the most frequent etiological agents in VAP [4]. Initial treatment decisions sought to identify clinical clues to suggest an etiology for VAP, such as invasive procedures, length of stay, prior antimicrobial use, immunosuppressive states and ecological flora data [4]. The decision to consider MRSA and vancomycin prescription during initial treatment takes into account the presence of diabetes mellitus, head trauma [5], comorbid conditions and local microbiological patterns of the ICU. In this context, the tracheal aspirate Gram stain can be used to guide initial empiric antimicrobial therapy. This rapid test could prevent the initiation of inappropriate therapy when culture results are not available. However, several studies attempting to determine its precision in VAP management have shown conflicting results [6–11].

In our hospital, to restrict antibiotic overuse and prevent the induction of multidrug-resistant organisms and the associated costs, a protocol created by the local Hospital Infection Control Committee precludes the use vancomycin for the initial empiric treatment of VAP in the absence of gram-positive cocci by tracheal aspirate.

The objective of this study is to evaluate the accuracy of Gram staining bacterioscopic analysis in tracheal aspirate samples to predict *Staphylococcus aureus presence or absence* in cultures of suspected VAP patients.

## Methods

This prospective single-center open cohort study enrolled patients admitted to a university teaching hospital ICU from December 2005 to December 2010. Three hundred ninety-nine patients suspected of having VAP by ATS IDSA criteria were included [4, 12]. Respiratory secretion samples were collected by tracheal aspirate for standard bacterioscopic analysis by Gram stain and culture. Patients who did not achieve the criteria for VAP by the Clinical Pulmonary Infection Score (CPIS) [2, 13] were excluded. All patient data were prospectively collected by researchers of the Infection Control Committee and registered in a database. All samples were Gram stained by the microbiology technicians and read by hospital microbiologists. Samples with more than 10 epithelial cells per field under 100x magnification were discarded and new material was collected for analysis. After evaluating bacterial smears by Gram stain, cultures were plated on chocolate agar and, if necessary, on blood agar, azide blood agar and McConkey agar. Analysis of the colonies was performed in order to quantify, identify the pathogen and its antimicrobial susceptibility profile. A culture was considered to be positive when culture density reached more than 10^5^ CFU/ml.

Statistical analysis: Data were analyzed using SPSS® 19 software (IBM, 2010). Relationships between the culture result and the Gram stain were tested with the chi-square test. Statistical significance was accepted at *P* < 0.05.

The present study was approved by the Hospital de Clínicas de Porto Alegre Research Ethics Committee. The committee waived requirements for informed consent because it was obtained in a previous study with the same cohort.

## Results

**Figure 1:**
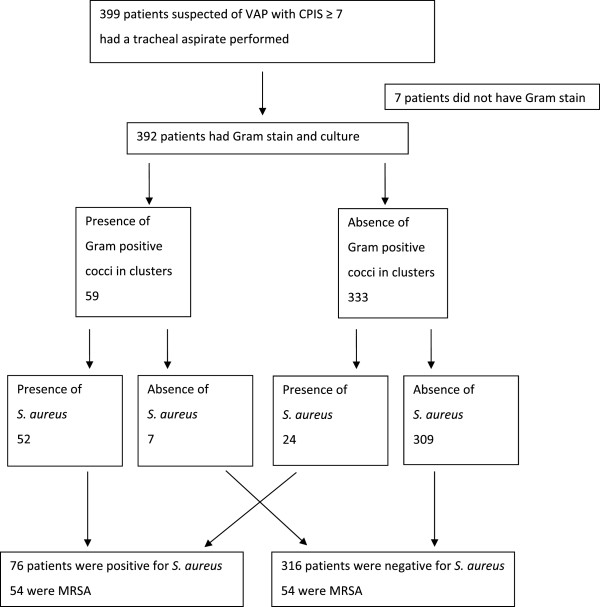
**The study design.** VAP, ventilator-associated pneumonia; CPIS, clinical pulmonary infection score; MRSA, methicillin- resistant *Staphylococcus aureus; S. aureus, Staphylococcus aureus*.

The study design is shown in Figure 1.

Samples of respiratory secretions collected by tracheal aspirates of 392 patients were analyzed by Gram stain and culture.

Gram positive cocci, including in clusters, chains and diplococci, were observed upon Gram staining of 148 (37.7%) samples.

Gram positive cocci in clusters were identified in 59 (15.1%) samples, and 52 (88.1%) of these contained *Staphylococcus aureus* isolated from the cultures.

Cross tabulation of the Gram stain and *Staphylococcus aureus* results in tracheal aspirate cultures is shown in Table 1.

**Table 1:** **Cross tabulation of the Gram stain and** ***Staphylococcus aureus*** **in the tracheal aspirate**

		Staphylococcus	Aureus	
		Yes	No	Total
Gram Positive	Yes	52	7	59
in clusters	No	24	309	333
	Total	76	316	392

The accuracy of the Gram stain in predicting *Staphylococcus aureus* in tracheal aspirate samples is shown in Table 2.

**Table 2:** **Accuracy of Gram stain in predicting** ***Staphylococcus aureus*** **in the tracheal aspirate**

Sensitivity	68.4
Specificity	97.8
Positive Predictive Value	88.1
Negative Predictive Value	92.8
Positive Likelihood Ratio	31.1
Negative Likelihood Ratio	0.3
Pre-Test Probability	19.4
Post-Test Probability – Positive	88.2
Post-Test Probability - Negative	7.2

Pearson Chi Square *p* = 0.0001.

When Gram-positive cocci were arranged in clusters, the sensitivity was 68.4%, specificity 97.8%, positive predictive value 88.1% and negative predictive value 92.8% for predicting the presence of *Staphylococcus aureus* in culture (*p* < 0.001).

Twenty eight (7.1%) samples contained Gram positive cocci in chains, and five of these contained *Staphylococcus aureus* isolated in culture. There was no statistical correlation between the presence of gram positive cocci in chains and the growth of *Staphylococcus aureus* in culture.

From all *Staphylococcus aureus* samples identified, 71.1% were methicillin-resistant.

## Discussion

In our study, we found a Positive Predictive Value of 82.1% and a Negative Predictive Value of 92,8% for the Gram stain in identification of *Staphylococcus aureus* in tracheal aspirate samples.

The high Negative Predictive Value observed in this study is in accordance with the results of Blot et al. [6]. When considering microbiologically confirmed VAP, these authors found that the sensitivity and specificity of the Gram stain examination were 89% and 56%, respectively, for the tracheal aspirate. The negative and positive predictive values of Gram stain examination for tracheal aspirate were 90% and 53%, respectively. Their results strongly suggest that when the Gram stain examination of tracheal aspirate is negative, the diagnosis of VAP is very unlikely. Similarly, Fagon et al. [14] demonstrated that Gram stain examination of the tracheal aspirate showed a good sensitivity (88,9%) but lacked specificity (59.6% false positives) in the diagnosis of VAP.

Other authors describe different results. Namias et al. [9] showed poor overall correlation between Gram positive cocci with the aspirate culture results in a study in which the Gram stain was used to guide empiric antibiotic treatment of pneumonia in surgical ICU patients. Tetenta and Metersky [11] found that the Gram stain had a sensitivity of 68%, a specificity of 72%, a negative predictive value of 80% and a positive predictive value of 59% for *Staphylococcus aureus*. However, the authors did not attempt to correlate the Gram stain findings with the presence of VAP. Therefore, some of these patients may have had low level colonization but not *Staphylococcus aureus* pneumonia.

Considering these results, providers are challenged to determine the level of acceptable risk associated with misdiagnosing a condition that has a mortality ranging between 25 to 76% [3, 4]. It is well established that early and appropriate empirical treatment reduces mortality [15–18]. The results of culture tests usually take 72 hours to become available, which could represent an unnecessary delay in determining the appropriate treatment.

Some evidence demonstrates that waiting for culture results may have important consequences for the patient. Iregui et al. [16] demonstrated that patients who met VAP diagnostic criteria and waited for culture results to initiate treatment experienced significant delays in receiving antibiotics. The mean time interval until the administration of antibiotic treatment was 28.6 h among patients who waited for culture results, compared to 12.5 h for all other patients (p < 0.001). Delayed administration of antibiotics was an independent risk factor for mortality (odds ratio, 7.68; 95%CI, 4.50 -13.09; p < 0.001).

Luna et al. [18] demonstrated that when adequate antibiotic therapy is initiated very early (before performing bronchoscopy), the mortality rate is reduced (38%) when compared to inadequate or absent therapies (91%) (p < 0.001). When patient treatment regimens were improved to adequate antibiotic therapy, mortality was comparable to those who continued to receive inadequate therapy based on BAL results. Kollef and Ward [17] showed that changing or modifying initially inadequate antibiotic treatment did not improve patient outcomes. The hospital mortality rate of patients who have their antibiotic therapy changed or whose therapy started following culture was 61%, while the mortality rate of patients who did not experience any changes in their antibiotic management was 33%. These treatment modifications likely occurred too late in the course of the illness to have a beneficial effect.

Rello and coworkers [19] also found a significant increase in mortality as a result of inappropriate early antibiotic therapy (37 versus 15%) despite guided changes in therapy after the results of cultures.

Based on these reports, it seems unquestionable that antibiotic treatment must be started early and that late escalation of treatment offers no clear advantage. Currently, Gram staining could be an useful tool if it provides accurate results. In this context, our findings demonstrate that to rule out the presence of *Staphylococcus aureus*, the Gram stain offered a Negative Predictive Value of 92.8%. Therefore, 7.2% of patients with *Staphylococcus aureus* would not be covered by an empiric treatment that precludes the use of vancomycin when there is not identification of Gram-positive cocci in clusters in the tracheal aspirate. The microbial ecology of the unit and clinical conditions should also be considered in the therapeutic decision. In settings with high incidence of MRSA infections, avoidance of empirical vancomycin usage increases the risk of inappropriate treatment.

Our study has some limitations. It is a single center study, and we did not include patients suspected of VAP with negative cultures. Similar to other studies, many samples of the lower respiratory tract were obtained from patients who had received previous or current antibiotics, potentially influencing the culture results. However, to establish the Positive Predictive Value and Negative Predictive Value, it is necessary to understand the true prevalence of infection, which requires a patient population with microbiologically confirmed VAP. Another limitation is the accuracy of clinical diagnosis of VAP with CPIS ≥ 7 confirmed by positive culture and the 2005 ATS IDSA diagnosis guidelines used in this study, which have been recently updated [20]. The prospective nature of our study is an advantage.

## Conclusions

Gram staining of the tracheal aspirate can rule out the presence of *Staphylococcus aureus* in patients with a clinical diagnosis of VAP with a 92.8% Negative Predictive Value. Therefore, 7.2% of patients with *Staphylococcus aureus* would not be protected by an empiric treatment that precludes the use vancomycin when Gram-positive cocci in clusters are not identified in the tracheal aspirate.

## Abbreviations

CFU: Colony-forming unit
CPIS: Clinical pulmonary infection score
ICU: Intensive care unit
MRSA: Methicillin-resistant *Staphylococcus aureus*
S. aureus: Staphylococcus aureus
TA: Tracheal aspirate
VAP: Ventilator-associated pneumonia.
